# MiR-122 Reverses the Doxorubicin-Resistance in Hepatocellular Carcinoma Cells through Regulating the Tumor Metabolism

**DOI:** 10.1371/journal.pone.0152090

**Published:** 2016-05-03

**Authors:** Chenwei Pan, Xiaodong Wang, Keqing Shi, Yi Zheng, Jie Li, Yongping Chen, Lingxiang Jin, Zhenzhen Pan

**Affiliations:** 1 Department of Infectious Disease, The Second Affiliated Hospital & Yuying Children’s Hospital of Wenzhou Medical University, Wenzhou, China; 2 Department of Infectious Disease, The First Affiliated Hospital of Wenzhou Medical University, Wenzhou, China; Taipei Medicine University, TAIWAN

## Abstract

Doxorubicin (DOX) is one of the most commonly used anticancer drugs in the treatment of hepatoma. However, acquired drug resistance is one of the major challenges for the chemotherapy. In this study, a down-regulation of miR-122 was observed in doxorubicin-resistant Huh7 (Huh7/R) cells compared with its parental Huh7 cells, suggesting miR-122 is associated with the chemoresistance. Meanwhile, luciferase reporter assay proved that the PKM2 is the target of miR-122, and we reported that the glucose metabolism is significantly up-regulated in Huh7/R cells. Importantly, overexpression of miR-122 in Huh7/R cells reversed the doxorubicin-resistance through the inhibition of PKM2, inducing the apoptosis in doxorubicin-resistant cancer cells. Thus, this study revealed that the dysregulated glucose metabolism contributes to doxorubicin resistance, and the inhibition of glycolysis induced by miR-122 might be a promising therapeutic strategy to overcome doxorubicin resistance in hepatocellular carcinoma.

## Introduction

Hepatocellular carcinoma (HCC) is one of the most common cancers worldwide, which is the third leading cause of cancer-related deaths [1]. Although surgery and liver transplants have high rate of cure for patients with early stage HCC, many patients are diagnosed when the disease has reached a stage beyond curative surgery [2]. In these cases, systemic chemotherapy is considered as an alternative option. Unfortunately, systemic chemotherapy is usually ineffective because of the resistance of cancer cells to chemotherapeutic agents, resulting in the high mortality from HCC [3]. Doxorubicin (DOX) is one kind of anthracycline drugs, which inhibits DNA/RNA synthesis by intercalation between base pairs of DNA strands, inducing apoptosis of tumor cells. Despite the doxorubicin is widely used for the treatment of HCC, the drug-resistance largely limited the clinical application of DOX [4,5]. Given this, combined treatment with some sensitizing agents is desirable to increase the anti-tumor effect and overcome the DOX-resistance.

MicroRNAs (miRNAs) are a class of small, endogenous, non-coding, single-stranded RNAs that regulate target-gene expression at post-transcriptional levels [6]. In recent years, miRNAs have emerged as the important class of gene regulator in cancer development [7], and studies have shown that about half of the human miRNAs are located in the cancer-associated genomic regions that are frequently amplified or deleted in cancers, suggesting that some miRNAs are involved in cell proliferation, differentiation, apoptosis, and drug resistance [8–9].

Current studies demonstrated that there exists major correlation between miRNAs and chemoresistance in multiple cancers. An *et al*. indicated that miR-23b-3p inhibited the autophagy mediated by ATG12 and HMGB2 and sensitized gastric cancer cells to chemotherapy [10]. Furthermore, several studies also demonstrated that the sensitivity of tumor cells to doxorubicin was associated with miRNAs. For example, overexpression of miR-181b in breast cancer induced doxorubicin-resistance by downregulating the pro-apoptotic protein of BIM [11]. MiR-125b sensitized the tumor cells to doxorubicin by targeting Mcl-1 [12]. Herein, we observed that miR-122 was down-regulated when the Huh7 cell line became doxorubicin-resistant. Furthermore, our data suggested that miR-122 plays an important role in doxorubicin therapy by targeting PKM2, which is a key regulator of tumor metabolism [13].

## Results

### MiR-122 is down-regulated in doxorubicin-resistant hepatocellular carcinoma cells

**Fig 1:**
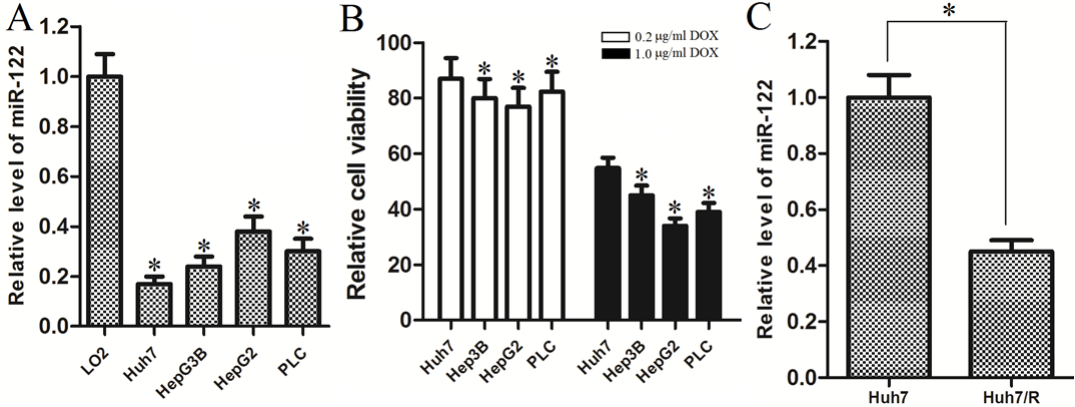
MiR-122 is down-regulated in hepatocellular carcinoma cell lines, and associated with doxorubicin resistance. (A) The expression of miR-122 was down-regulated in HCC cell lines compared with the normal hepatocytes. *p<0.05 vs. L-O2 cells, t test. (B) The cell viability of Huh7, Hep3B, HepG2, and PLC was measured by MTT assay after they were treated with 0.2 μg/ml doxorubicin or 1.0 μg/ml doxorubicin for 48 h. *p<0.05 vs. Huh7 cells, t test. (C) MiR-122 expression was further down-regulated in Huh7/R cells compared with its parental Huh7 cells. *p<0.05, t test.

To investigate the role of miR-122 in HCC, we measured the expression of miR-122 in multiple HCC cell lines. We found that the expression of miR-122 was significantly down-regulated in HCC cell lines (Huh7, Hep3B, HepG2 and PLC) compared with the L-O2 cell line which is the normal hepatocytes (Fig 1A), suggesting miR-122 function as a tumor suppressor in HCC. As the Huh7 was the most insensitive cell line to doxorubicin treatment (Fig 1B), we chose it as the cell model for the study of DOX-resistance in HCC. Interestingly, we found that the miR-122 level was further down-regulated when the Huh7 cells became doxorubicin-resistant (Fig 1C). All these results suggest that miR-122 is a tumor suppressor, and associated with doxorubicin resistance in HCC.

### Overexpression of miR-122 resensitizes Huh7/R cells to doxorubicin inducing cytotoxicity

**Fig 2:**
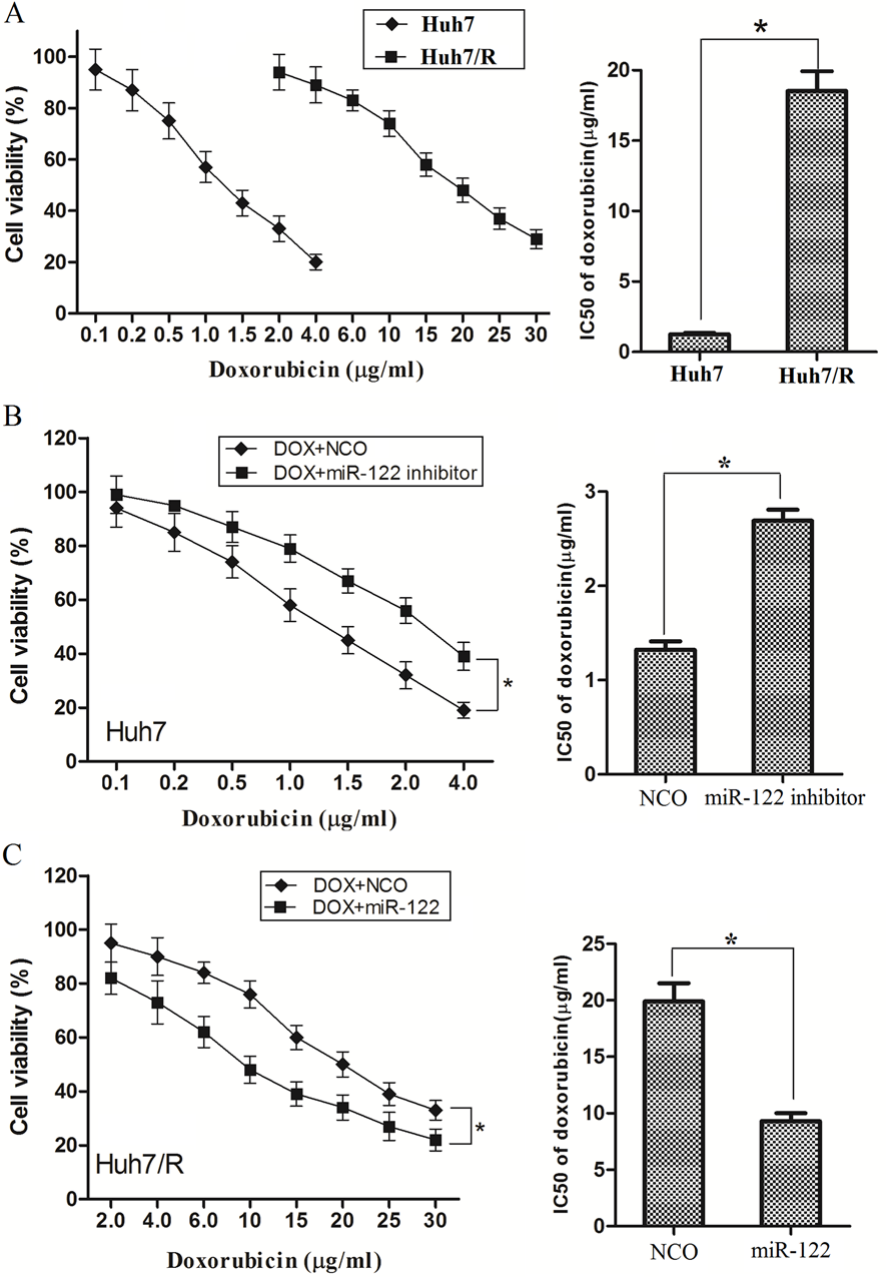
Overexpression of miR-122 resensitizes Huh7/R cells to DOX. (A) Huh7 and Huh7/R cells were treated with different concentrations of doxorubicin for 48 h, and cell viability was determined by MTT assay. The IC50 was determined according to the survival curves. *p<0.05, t test. (B) Huh7 cells were transfected with miR-122 inhibitors or NCO for 24 h followed by the treatment of DOX at different concentrations for another 48 h followed by cell viability analysis. The IC50 was determined according to the survival curves. *p<0.05, t test. (C) Huh7/R cells were transfected with miR-122 mimics or NCO for 24 h followed by the treatment of DOX at different concentrations for another 48 h followed by cell viability analysis. The IC50 was determined according to the survival curves. *p<0.05, t test.

To verify the resistance, parental cells (Huh7) and doxorubicin-resistant Huh7 cells (Huh7/R) were treated with DOX at different concentrations for 48 h. As we expected, cell viability assays showed that Huh7/R cells could tolerate much higher concentrations of DOX (The transfection efficiency of miR-122 mimics and miR-122 inhibitor was shown in S1 Fig). Comparing with the Huh7 cells, the IC50 for DOX was fifteen fold higher in the resistant cells (18.51μg/ml) compared with the parent cells (1.25μg/ml) (Fig 2A). Next, we examined whether the overexpression of miR-122 was capable of sensitizing Huh7/R cells to DOX. Comparing with negative control, the knockdown of miR-122 by its inhibitors significantly decreased the sensitivity of Huh7 cells to DOX (Fig 2B). In contrast, overexpression of miR-122 markedly enhanced DOX induced cell cytotoxicity in Huh7/R cells (Fig 2C). Our date strongly suggested that miR-122 is negative correlated with DOX resistance in HCC.

### PKM2 is a direct target of miR-122 in Huh7/R cells

**Fig 3:**
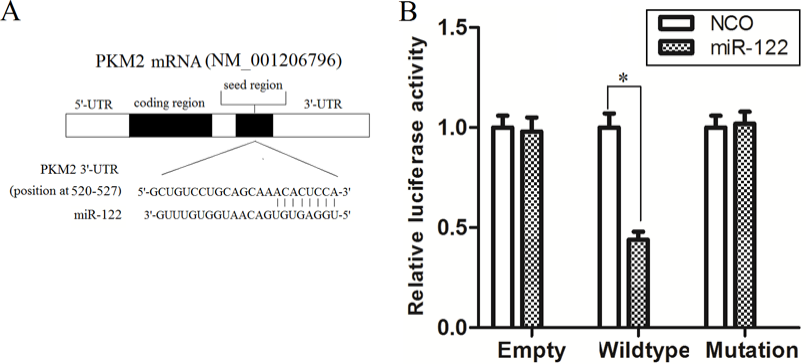
PKM2 is a direct target of miR-122. (A) Putative miR-122 binding sequence in the 3′-UTR of PKM2 mRNA. (B) Huh7/R cells were co-transfected with luciferase reporter plasmids, pRL-TK vector and miR-122 (or NCO) using lipofectamine 2000 reagent. After transfection for 48 h, luciferase activities were measured by a dual luciferase reporter assay. The pRL-TK vector was used as an internal control. The results were determined as relative luciferase activity (Firefly LUC/Renilla LUC). *p<0.05, t test.

To determine how miR-122 enhances the cytotoxicity of DOX to Huh7/R, we next investigated the mechanism of miR-122 in DOX resistance. By searching miRNA database (TargetScan, http://www.targetscan.org) for the prediction of miR-122 targets that may possibly contribute to doxorubicin resistance, we found that PKM2, which has been reported to be associated with drug-resistance and is the main form of pyruvate kinase isoenzyme (PKM) in cancers [14–15], might be a target for miR-122, whose 3’-UTR contains a highly conserved binding site for miR-122 (Fig 3A). To validate whether PKM2 is an actual target of miR-122, we performed Luciferase reporter analysis by co-transfecting the Huh7/R cells with miR-122 mimics (or NCO) and pMIR reporter plasmid which contains wild-type (or mutant) miR-122 binding sites. The overexpression of miR-122 significantly decreased the luciferase activity of the reporter with wild-type 3’-UTR of PKM2 in Huh7/R cells. However, the luciferase activity of the mutant (or empty) reporter in the presence of miR-122 was almost unaffected (Fig 3B). Taken together, our results demonstrated that PKM2 is a direct target of miR-122 in Huh7/R cells.

### PKM2 is overexpressed in doxorubicin-resistant Huh7 cells

**Fig 4:**
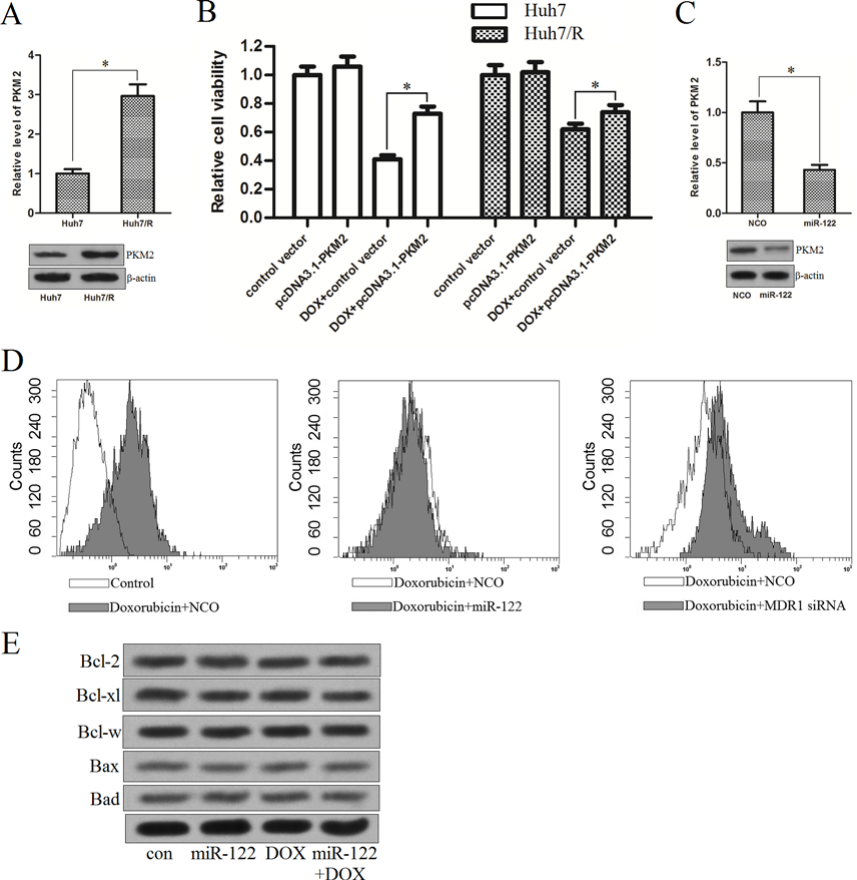
PKM2 is overexpressed in doxorubicin-resistant Huh7 cells. (A) The expression of PKM2 was detected by qPCR and western blot analysis in Huh7/R cell line and its parental Huh7 cell line. *p<0.05, t test. (B) The Huh7 and Huh7/R cells were transfected with PKM2 recombinant vector (2 μg/ml) for 24 h. The Huh7 and Huh7/R cells were then treated with 1.5 μg/ml DOX and 15 μg/ml DOX for another 48 h, respectively. Cell viability was measured by MTT assay. *p<0.05, t test. (C) Transfection of miR-122 decreased the PKM2 level in Huh7/R cells. *p<0.05, t test. (D) Transfection of miR-122 didn’t influence the doxorubicin accumulation in Huh7/R cells. (E) Western blot analysis was performed to evaluate the expression of Bcl-2, Bcl-xl, Bcl-w, Bax, and Bad in Huh7/R cells treated with miR-122 or doxorubicin.

Since we demonstrated that PKM2 is directly regulated by miR-122 in Huh7/R cells, we next investigated the correlation between the PKM2 and the doxorubicin-resistance in HCC cells. As shown in Fig 4A, we found the expression of PKM2 was up-regulated in Huh7/R cells at both mRNA level and protein level compared with the parental Huh7 cells, suggesting the overexpression of PKM2 may be essential in doxorubicin-resistance. To confirm this hypothesis, we transfected the Huh7 and Huh7/R cells with pcDNA3.1-PKM2 which made the PKM2 overexpress in them (The transfection efficiency of pcDNA3.1-PKM2 was shown in S1 Fig). We found the enforced expression of PKM2 significantly inhibited the DOX-induced cell death in Huh7, whereas the effect of pcDNA3.1-PKM2 on DOX-treated Huh7/R cells was slight (Fig 4B). These results indicated that PKM2 acted important role in doxorubicin-resistance, which was adequate in DOX-resistant HCC cells. In addition, we found that compulsive expression of miR-122 significantly down-regulated the PKM2 level (Fig 4C). To explore the mechanisms why miR-122 reversed the doxorubicin resistance in Huh7/R cells, we measured the DOX-accumulation in Huh7/R cells transfected with miR-122 mimics, taking MDR1 (encoding P-glycoprotein) siRNA as the positive control to increase the accumulation of DOX [16]. As shown in Fig 4D, the transfection of MDR1 siRNA significantly increased the accumulation of doxorubicin as previous described. However, the DOX accumulation in Huh7/R cells was not influenced after the miR-122 transfection, suggesting that there exists another pathway for the synergism of miR-122 and DOX. In addition, the expression of Bcl-2 family proteins was not altered in Huh-7/R cells treated with miR-122 or DOX (Fig 4E). We therefore inferred that the exogenous miR-122 enhances the anti-cancer effect of doxorubicin by down-regulating the PKM2 level in doxorubicin-resistant HCC cells, which is the key regulator of tumor metabolism.

### Huh7/R cells exhibit increased glucose metabolism via miR-122-PKM2 pathway

**Fig 5:**
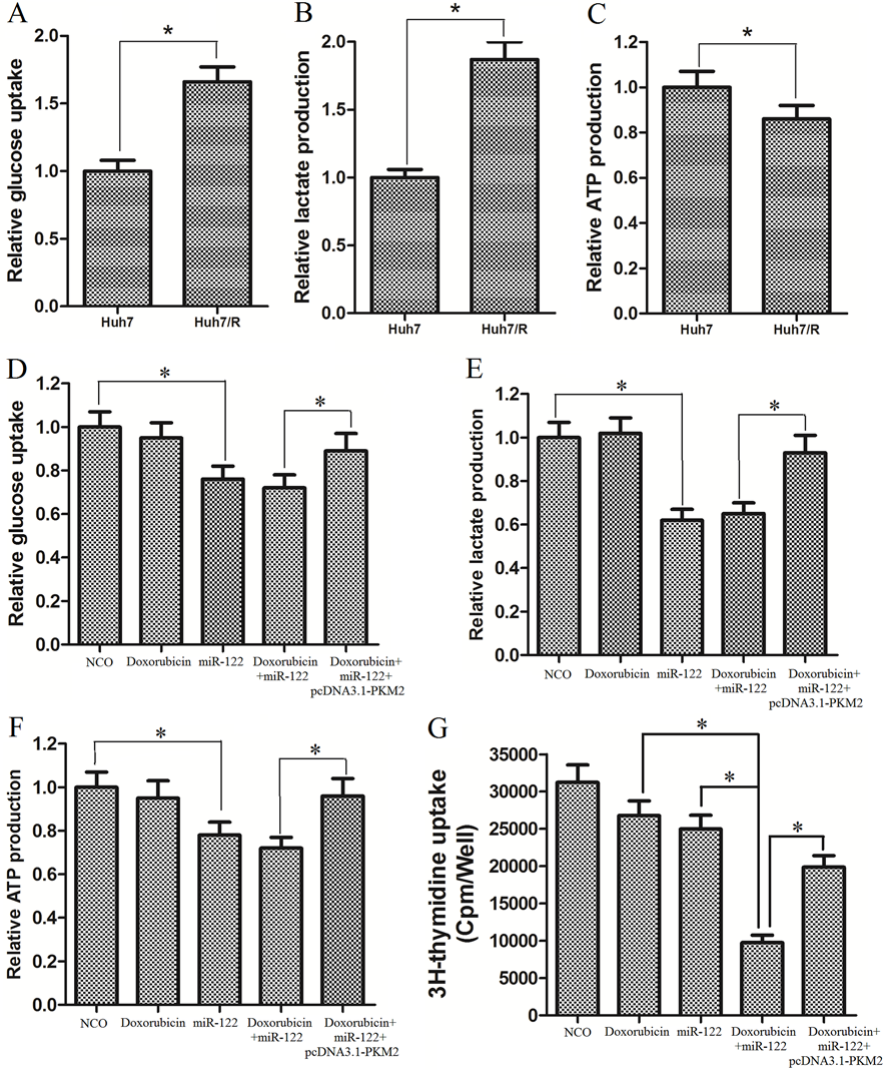
Huh7/R cells exhibit increased glucose metabolism via miR-122-PKM2 pathway. (A) Glucose uptake of Huh7/R cells was increased. *p<0.05, t test. (B) Lactate production in Huh7/R cells was increased. *p<0.05, t test. (C) ATP production in Huh7/R cells was decreased. *p<0.05, t test. (D) MiR-122 but not doxorubicin significantly decreased the glucose uptake in Huh7/R cells. *p<0.05, t test. (E) MiR-122 but not doxorubicin significantly decreased the lactate production in Huh7/R cells. *p<0.05, t test. (F) MiR-122 but not doxorubicin further reduced the ATP production in Huh7/R cells. *p<0.05, t test. (G) Overexpression of miR-122 inhibited the proliferation of Huh7/R cells, and significantly enhanced the anti-proliferation effect of DOX on Huh7/R cells. *p<0.05, t test.

Previous studies revealed that tumor cells exhibit altered metabolism which is linked to the chemoresistance [17]. To study whether the doxorubicin-resistant Huh7 cells exhibit altered aerobic glycolysis, we measured several key glycolytic parameters, including glucose consumption, lactate production, and ATP production in the Huh7/R cells and their parental Huh7 cells. As shown in Fig 5A and 5B, Huh7/R cells consumed more glucose as well as releasing more lactate than their parental cells. However, the resistant cells produced less ATP rather than the parental cells (Fig 5C). These results suggested that dysregulated glucose metabolism was correlated with doxorubicin resistance in HCC cells. To investigate the role of miR-122 in glucose metabolism and the mechanism of reversing the DOX-resistance in Huh7/R cells, we treated the cells with miR-122 mimics and DOX, and then the glycolytic parameters were measured. Consistent with our inference, we found that miR-122 but not doxorubicin significantly decreased the glucose uptake and lactate production. Importantly, the biological effect of miR-122 was inhibited by transfecting with pcDNA3.1-PKM2 in Hhu7/R cells (Fig 5D and 5E). On the other hand, the ATP production was further reduced caused by miR-122 tranfection-inducing down-regulation of PKM2 (Fig 5F). Previous studies demonstrated that the high glycolytic capacity is crucial for proliferation of cancer cells [18]. We therefore studied the proliferation of DOX-resistant Huh7/R cells treated with DOX and miR-122. As shown in Fig 5G, we found overexpression of miR-122 inhibited the proliferation of Huh7/R cells, and significantly enhanced the anti-proliferation effect of DOX on Huh7/R cells.

### MiR-122 sensitized DOX-resistant Huh7 cells to doxorubicin inducing cell death via miR-122-PKM2 pathway

**Fig 6:**
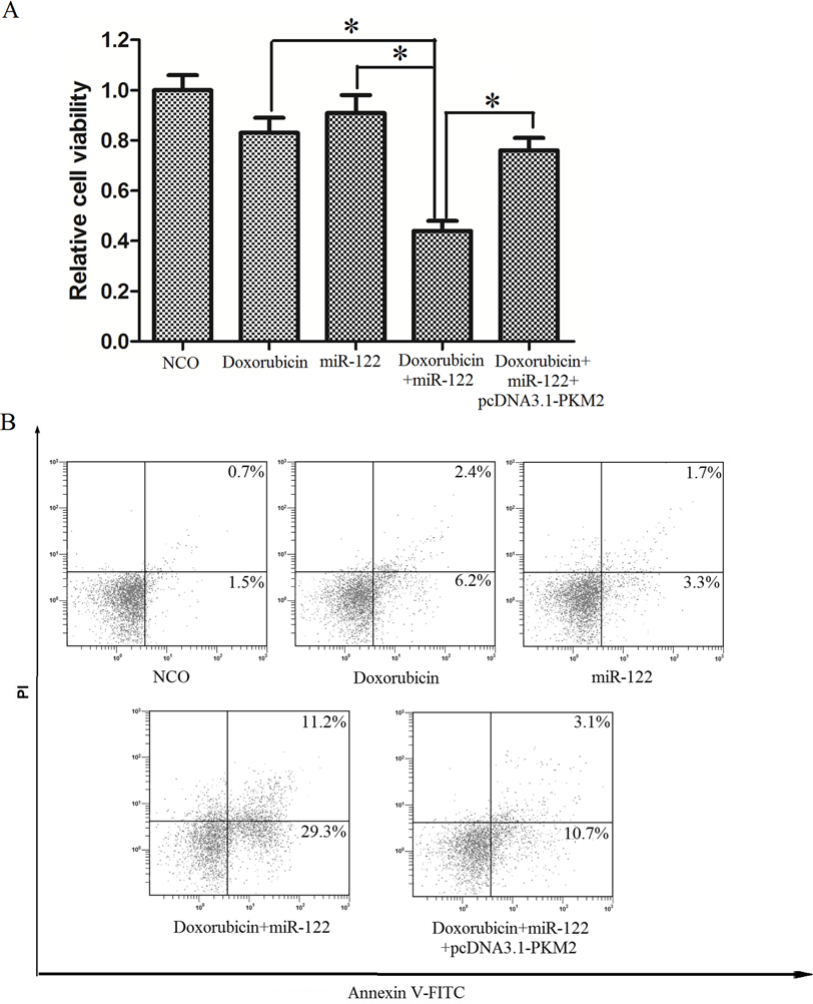
MiR-122 sensitized Huh7/R cells to doxorubicin inducing cell death via miR-122-PKM2 pathway. (A) Huh7/R cells were transfected with miR-122 mimics as well as PKM2 recombinant vector for 24 h, and then the cells were treated with doxorubicin (10 μg/ml) for another 48 h. Cell viability was measured by MTT assay. *p<0.05, t test. (B) Huh7/R cells were transfected with miR-122 mimics as well as PKM2 recombinant vector for 24 h, and then the cells were treated with doxorubicin (10 μg/ml) for another 24 h. Cell apoptosis was measured using Annexin V/PI staining. (C) Cleavage of caspase-3 and PARP was evaluated by western blot analysis.

To investigate the role of PKM2 in the sensitization of miR-122 to DOX, we measured the cell viability of Huh7/R cells by MTT assay. As described above, Huh7/R cells were resistant to DOX alone treatment, and the transfection of miR-122 significantly enhanced the cytotoxicity of DOX to Huh7/R cells. Importantly, the overexpression of PKM2 by vector significantly inhibited the synergistic effect of miR-122 on DOX-inducing cell death (Fig 6A), suggesting the key role of miR-122-PKM2 pathway in doxorubicin based therapy. Since the anti-cancer effect of doxorubicin is dependant on the apoptosis induction resistance in Huh7/R cells through inducing apoptosis [19], we next studied the role of miR-122-PKM2 pathway in DOX-inducing apoptosis in Huh7/R cells. As we observed, miR-122 transfection significantly enhanced the apoptosis (Fig 6B) and cleavage of caspase-3 and PARP (Fig 6C) in Huh7/R cells treated with DOX, which could be inhibited by co-transfecting with pcDNA3.1-PKM2. In conclusion, all of the results showed in this paper strongly suggested the important role of miR-122 in reversing the doxorubicin-resistance in hepatocellular carcinoma cells by regulating the tumor metabolism through the miR-122-PKM2 pathway.

## Discussion

MiR-122 is one of the most frequent miRNAs isolated in the liver, which was not detected in any of the other tissues [20]. Studies have demonstrated that miR-122 acts as a tumor suppressor in HCC. Coulouarn, *et al*. revealed that miR-122 is down-regulated in HCC cells, and reintroduction of miR-122 into HCC cells can reverse the tumorigenic properties of these cells [21]. Tsai, *et al*. demonstrated that Down-expression of miR-122 was significantly correlated with proliferation and metastasis by targeting ADAM17 (a disintegrin and metalloproteinase 17) [22]. Interestingly, studies also showed overexpression of miR-122 could improve the sensitivity of HCC cells to the common chemotherapeutic compound, such as sorafenib [23]. In this study, we confirmed that miR-122 is a tumor suppressor in HCC, which was associated with the resistance of doxorubicin-based therapy.

Tumorigenesis is characterized by the alteration of glycolytic metabolism as well as the changes of the glycolytic isoenzyme, which gives several advantages to cancer cells [24]. Instead of oxidative phosphorylation, tumor cells showed high glycolytic rate with production of lactate even in an oxygen-rich condition [25]. In tumor cells, glucose is used for anabolic processes preferentially rather than oxidative phosphorylation, resulting in the high level of NADPH and ribose-5-phosphate for the synthesis of nucleotides, which contributed to the chemoresistance [26]. To regulate the balance between glycolytic energy regeneration [producing adenosine triphosphate (ATP) for energy supply] and the synthesis of cell building blocks (producing protein, lipid and nucleic acid for proliferation and chemoresistance) in tumor cells, the pyruvate kinase isoenzyme M2 (PKM2) is usually overexpressed [27–28]. Previous studies have reported that PKM2 was involved in cell growth in various tumors and drug-resistance. For example, the increased PKM2 expression was associated with later stage and lymph metastasis of the colorectal cancer cells, which also promoted the oxaliplatin resistance [29–30]; In HCC, the PKM2 also promoted metastasis by recruiting the myeloid-derived suppressor cells and indicated poor prognosis [31].

In this study, we observed that the Huh7/R cells showed increase of glucose uptake and lactate production compared with Huh7 cells. Interestingly, however, the ATP production was reduced. We explained that the overexpression of PKM2 in Huh7/R reconstructed the balance between the energy regeneration and the biosynthesis, and the increase of glucose was used to produce cell building blocks instead of producing the ATP. Furthermore, we indicated that miR-122 reversed the doxorubicin-resistance via regulating the expression of PKM2, without influencing the intracellular DOX accumulation and the expression of Bcl-2 family proteins in Huh7/R cells. Since the balance between energy production and glycolysis was broken by the knockdown of PKM2 caused by miR-122 in Huh7/R, we found the glucose uptake, lactate release and ATP production was significantly decreased. And what’s more, the proliferation ability was also be interfered by miR-122, because the synthesis of cell building block was inhibited. Researches have demonstrated that the intracellular ATP levels are a pivotal determinant of chemoresistance in tumor cells, which is a switch to mitochondrial dysfunction-induced apoptosis [32–33]. The mechanisms of resistance to chemotherapy-induced cell death in cancer cells are included at least one of the following: enhancement of DNA damage repair, enhancement of drug inactivation, mutation of survival-related genes, dysregulation of growth factor signaling pathways, and/or activation of intracellular survival signaling [34–35]. All of these activities consume ATP. Our data proved that the down-regulation of PKM2 induced by miR-122 inhibited the DOX-resistant HCC cells to produce enough ATP which is needed for chemoresistant cells to survive under stress. On the other hand, doxorubicin exerts cytotoxicity by inhibits DNA/RNA synthesis, leading to the lethal DNA damage followed by the initiation of cell death through apoptosis pathway [36]. Consistent with these points, our study indicated that the introduction of miR-122 inhibited the expression of PKM2 and subsequent ATP production, lack of which facilitated the doxorubicin-induced apoptosis in DOX-resistant HCC cells.

In summary, the present study demonstrated a regulatory role of miR-122 in HCC metabolism, by targeting PKM2 that is involved in aerobic glycolysis. Our data presented the essential role of miR-122 in doxorubicin resistance, and the miR-122-PKM2 pathway may provide a novel therapeutic strategy for treatment of HCC.

## Materials and Methods

### Reagents and antibodies

Doxorubicin, 3-(4,5-dimethylthiazol-2-yl)-2,5-diphenyltetrazolium bromide (MTT), Annexin V-FITC Apoptosis Detection Kit was obtained from Sigma-Aldrich (USA). Antibodies for anti-human PKM2 and anti-human β-actin were purchased from Cell Signaling (USA). MiR-122 mimics, miR-122 inhibitor, MDR1 siRNA and negative control oligonucleotide (NCO) were purchased from Genepharma Company (China), and the sequences were as follows: miR-122 mimics: 5'-UGGAGUGUGACAAUGGUGUUUG-3'; 2′-Omethyl modified miR-122 inhibitor: 5'-CAAACACCAUUGUCACACUCCA-3'; NCO: 5'-UGUGGAGUGUUAUGUCAGAGUG-3'; MDR1 siNRA: 5'-GGAAAAGAAACCAACUGUCUU-3'.

### Cell Culture

The human embryo liver cell line (L-02) which is considered as the normal hepatocytes [37], and the HCC cell lines including Huh7, Hep3B, HepG2 and PLC were purchased from the Institute of Biochemistry and Cell Biology, Chinese Academy of Sciences (Shanghai, China). The cell lines were cultured in the DMEM basic medium (Gibco, USA) containing 10% fetal bovine serum (FBS, Gibco, USA) at 37°C in a humidified 5% CO2 incubator. Huh7/R (DOX-resistant Huh7) cells were established by gradual exposure of Huh7 cells to increasing concentrations of doxorubicin in regular cell culture conditions for the selection of resistant cells [38]. Briefly, the Huh7 cells were initially treated with DOX at 0.2 μg/ml for 2 months, and then the DOX concentration was increased every 3 weeks by 0.04 μg/ml up to a final concentration of 0.6 μg/ml. The Huh7/R cells were exposed to DOX over a time period of 12 months. Importantly, the DOX would be removed from the culture medium for 2 weeks before the Huh7/R cells were performed to the following experiments.

### Quantitative RT-PCR

Total RNA was extracted from cell lines using Trizol reagent (Invitrogen, USA), and the cDNA was synthesized using M-MLV Reverse Transcriptase (Invitrogen, USA) following the manufacturer's instructions. For qRT-PCR analysis of PKM2 and GAPDH mRNA expression, 1μg of total RNA was reverse-transcribed to cDNA with oligod(T) (Invitrogen). The primer sequences are as follows: PKM2 F: 5’-GCCTGGCGCCCATTACCA-3’, R: 5’-CCCACTGCAGCACTTGAAG-3’; GAPDH F: 5′-CACTCCTCCACCTTTGA-3′, GAPDH R: 5′-CCACCACCCTGTTGCTG-3′. The relative expression level was determined using the 2^-△△CT^ analysis method, where GAPDH was used as an internal reference. For analysis of miR-122 expression by qRT-PCR, the specific stem-loop RT primers [39] were used for reverse transcription reaction as the following: miR-122: 5′-CTCAACTGGTGTCGTGGAGTCGGCAAT TCAGTTGAGCAAACACC-3′; U6: 5′-AACGCTTCACGAATTTGCGT-3′. The U6 snRNA was used as the internal reference to determine the relative expression of PKM2. PCR analysis was performed by ABI PRISM 7900 Sequence Detection System.

### Vector construction and luciferase assay

The PKM2 expression vector was constructed by PCR amplification of the open reading frame of PKM2 gene without the 5’- and 3’-UTR region. The amplified fragment was cloned into pcDNA3.1 (Clontech, USA), named pcDNA3.1-PKM2. To construct PKM2-3’UTR plasmid, a wild-type 3’-UTR fragment of human PKM2 mRNA (GenBank accession No. NM_001206796) containing the putative miR-122 binding sequence was amplified by PCR and cloned into the pMIR-REPORT™ miRNA Expression Reporter Vector (Life Technologies, USA) which is the downstream of the luciferase reporter gene. The recombinant plasmid was named pMIR-wildtype. The mutations of pMIR plasmid was generated by mutating the complementary site for the seed region of miR-122 (ACACUCCA to ACAUCCCA) by using site-directed mutagenesis kit (Takara, Japan) based on the wild-type plasmid, named pMIR-mutation. Huh7/R cells were transfected with the recombinant pMIR plasmid (2 μg/ml) and Renilla luciferase pRL-TK vector (Promega USA, 100 ng/ml) using lipofectamine 2000 reagent (Invitrogen, USA) according to the manufacturer’s protocol. Cells were collected 48 h after transfection and analyzed using the Dual-Luciferase Reporter System (Promega, USA) according to the manufacturer's instructions. Firefly luciferase activity was normalized to the Renilla luciferase activity, and shown as the ratio between the cells transfected with miR-122 and cells transfected with NCO.

### Measurement of cell viability by MTT assay

Cells were seeded onto 6-well plate, and then the pcDNA3.1-PKM2 (2 μg/ml), RNA oligonucleotides (NCO, miR-122 mimics, miR-122 inhibitors or MDR1 siNRA, 50 nM) were transfected on the next day. After 24 h post-transfection, cells were trypsinized and seeded onto 96-well plates at a density of 5,000 cells/well with different concentrations of DOX incubating for another 48 h. And then, a MTT assay was performed to measure the cell viability. DOX concentrations leading to 50% cell death (IC50) were determined by the MTT-dependent cell viability assay.

### Proliferation assay

Cells were seeded onto 6-well plate, and then the pcDNA3.1-PKM2 (2 μg/ml), RNA oligonucleotides (NCO, miR-122 mimics, 50nM) were transfected on the next day. After 24h post-transfection, the cells were washed with PBS and incubated with fresh DMEM medium with 10% FBS in the absence or presence of DOX (10 μg/ml) for 18 h. ^3^H thymidine incorporation assay was used to determine the cell proliferation during the last 6 hours of incubation as previously described [40].

### Doxorubicin accumulation

Cells were seeded onto 6-well plate, and then the RNA oligonucleotides (NCO, miR-122 mimics, MDR1 siRNA, 50 nM) were transfected on the next day. After 24 h post-transfection, the cells were washed with PBS and incubated with fresh DMEM medium with 10% FBS in the absence or presence of DOX (10 μg/ml). After 2 h incubation, cells were washed three times with PBS and the mean fluorescence intensity of intracellular DOX was determined using flow cytometry at an excitation wavelength of 488 nm and an emission wavelength of 575 nm.

### Glucose uptake and lactate product assay

Cells were seeded onto 6-well plate, and then the pcDNA3.1-PKM2 (2 μg/ml), RNA oligonucleotides (NCO, miR-122 mimics, 50 nM) were transfected on the next day. After 24 h post-transfection, the cells were washed with PBS and incubated with fresh DMEM medium with 10% FBS in the absence or presence of DOX (10 μg/ml) for 24 h. The relative glucose uptake was measured using an Amplex Red Glucose/Glucose Oxidase assay kit (Molecular Probes, USA) according to the manufacturer’s protocol. Similarly, the lactate production in the medium was detected using a Lactate assay kit (BioVision, USA). The results were normalized to the NCO group.

### ATP measurement

Cells were seeded onto 6-well plate, and then the pcDNA3.1-PKM2 (2 μg/ml), RNA oligonucleotides (NCO, miR-122 mimics, 50 nM) were transfected on the next day. After 24 h post-transfection, the cells were washed with PBS and incubated with fresh DMEM medium with 10% FBS in the absence or presence of DOX (10 μg/ml) for another 24 h. After incubation, the cells were harvested and lysed in 1% NP40 solution, and when the ATP in the lysates combined with the luciferin/luciferase enzyme complex, a reaction which produces light occurs. The relative light units were detected by Multimode Detector (DTX 880, Beckman Coulter).

### Western blot analysis

The whole cell extracts were prepared by lysing cells in RIPA buffer (Cell Signaling, USA). Protein concentration was determined using a BCA Protein Assay Kit (Pierce, USA), and 50 μg of protein was separated by 12.5% SDS-PAGE and transferred onto PVDF membranes. The membranes were then incubated by the PKM2 anti-bodies, and the β-actin expression was evaluated as a control for protein loading.

### Apoptosis assay

Cells were seeded onto 6-well plate, and then the pcDNA3.1-PKM2 (2 μg/ml), RNA oligonucleotides (NCO, miR-122 mimics, 50 nM) were transfected on the next day. After 24 h post-transfection, the cells were washed with PBS and incubated with fresh DMEM medium with 10% FBS in the absence or presence of DOX (10 μg/ml) for another 24 h. After incubation, cells were incubated with Annexin V and Propidium Iodide (PI) for 15 min at room temperature according to the manufacturer's instructions and analyzed using flow cytometry (Becton Dickinson, USA).

### Statistical analysis

Data analysis was performed with SPSS 12.0 statistical software, and expressed as mean ± SE. Differences were assessed by Student’s t test. *P*<0.05 was considered to be statistically significant. All of the date was derived from at least three independent experiments.

## Supporting Information

S1 Fig The transfection efficiency of RNA oligonucleotides and recombinant plasmids. **(A)** MiR-122 mimics or miR-122 inhibitors were transfected into Huh7/R cells or Huh7 cells, respectively. The expression of PKM2 was measured by qRT-PCR analysis. *p<0.05, t test. **(B)** Huh7/R cells were transfected with recombinant pcDNA3.1 plasmid carried with PKM2 gene. After 24h, the expression of PKM2 at mRNA level was measured by qRT-PCR analysis. *p<0.05, t test. **(C)** Huh7/R cells were transfected with recombinant pcDNA3.1 plasmid carried with PKM2 gene. After 24h, the expression of PKM2 at protein level was determined by western blot analysis.
(TIF)
