# Supplementary figures and images for: MiR-122 Reverses the Doxorubicin-Resistance in Hepatocellular Carcinoma Cells through Regulating the Tumor Metabolism
Source: PLoS One. 2016 May 3;11(5):e0152090. doi: 10.1371/journal.pone.0152090 (PMC4854441; doi:10.1371/journal.pone.0152090)

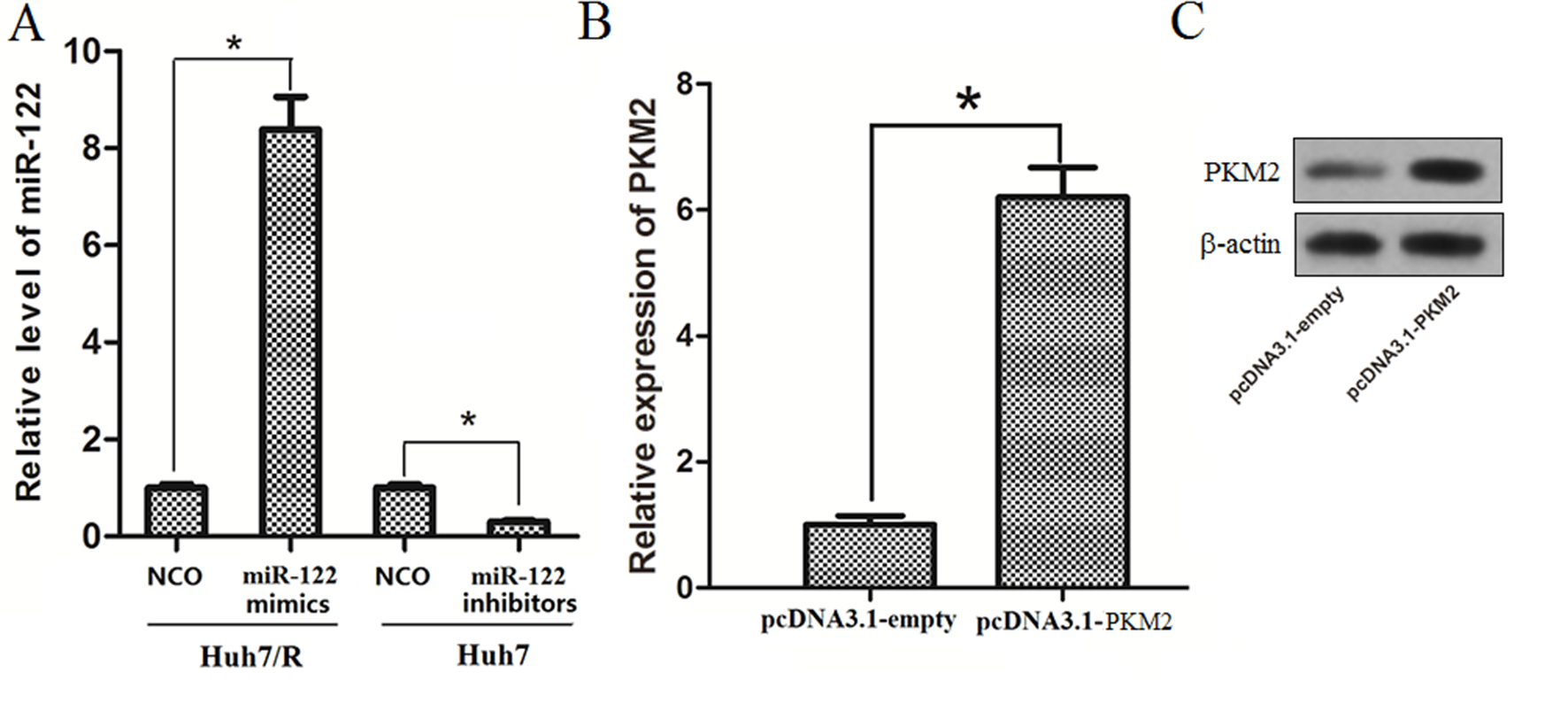

Supplement: S1 Fig — (A) MiR-122 mimics or miR-122 inhibitors were transfected into Huh7/R cells or Huh7 cells, respectively. The expression of PKM2 was measured by qRT-PCR analysis. *p<0.05, t test. (B) Huh7/R cells were transfected with recombinant pcDNA3.1 plasmid carried with PKM2 gene. After 24h, the expression of PKM2 at mRNA level was measured by qRT-PCR analysis. *p<0.05, t test. (C) Huh7/R cells were transfected with recombinant pcDNA3.1 plasmid carried with PKM2 gene. After 24h, the expression of PKM2 at protein level was determined by western blot analysis. (TIF) [file pone.0152090.s001.tif]
